# Gelatin Type A from Porcine Skin Used as Co-Initiator in a Radical Photo-Initiating System

**DOI:** 10.3390/polym11111901

**Published:** 2019-11-18

**Authors:** Andrea Cosola, Annalisa Chiappone, Cinzia Martinengo, Hansjörg Grützmacher, Marco Sangermano

**Affiliations:** 1 Department of Applied Science and Technology, Politecnico di Torino, Corso Duca degli Abruzzi 24, 10129 Turin, Italy; andrea.cosola@polito.it (A.C.); annalisa.chiappone@polito.it (A.C.); cinzia.martinengo@polito.it (C.M.); 2 Department for Chemistry and Applied Biosciences, ETH Zürich, Vladimir-Prelog-Weg 1, CH 8093 Zürich, Switzerland; hgruetzmacher@ethz.ch

**Keywords:** gelatin, PEGDA, hydrogel, camphorquinone, photopolymerization, radical photo-initiating system

## Abstract

In the present study, a different approach for the preparation of poly(ethylene glycol) diacrylate-gelatin (PEGDA-gelatin) hydrogels was investigated. Gelatin type A from porcine skin was used as the co-initiator of a radical photo-initiating system instead of the traditional aliphatic or aromatic amines. This became possible because, upon visible-light irradiation, the amine sequences within gelatin generate initiating free-radicals through the intermolecular proton transfer in a Norrish type II reaction with camphorquinone (CQ). PEGDA-gelatin hydrogels were prepared by visible-light-induced photopolymerization. The gelatin content in the precursor formulations was varied. The influence of gelatin on the kinetics of the photocuring reaction was investigated, and it was found that gelatin fastened the rate of polymerization at all concentrations. The covalent attachment of gelatin segments within the cross-linked hydrogels was evaluated by means of attenuated total reflectance-infrared spectroscopy (ATR-FTIR) spectroscopy after solvent extraction. The thermo-mechanical properties, as well as the swelling behavior and gel content, were also investigated.

## 1. Introduction

Hydrogels are three-dimensional polymeric networks that are able to absorb and retain large amounts of water without dissolving [1,2,3]. Due to their water content and tunable physico-chemical properties, they have been increasingly used in tissue engineering (TE) to mimic soft human tissues, and they have been proposed as scaffold materials in regenerative medicine to allow for cell attachment, growth and differentiation [4,5,6].

Both synthetic and natural polymers have been proposed as precursors for the production of hydrogels; while synthetic materials enable a greater control over chemical composition, gel behavior and mechanical properties, natural materials are preferable for their biocompatibility [7]. Among the synthetic precursors proposed in the literature (e.g., poly(ethylene glycol) (PEG), poly(acrylic acid) (PAA), polyacrylamide (PAAm), poly(vinyl alcohol) (PVA) and poly(N-vinyl pyrrolidone (PVP)), PEG-based hydrogels have been largely studied for biomedical applications such as the fabrication of scaffolds via photopolymerization [8]. PEG is a versatile hydrophilic polymer with two hydroxyl end groups that can be converted into functional groups, such as methacrylates or acrylates, in order to generate photocurable hydrogels [9]. However, even if PEG-hydrogels are largely used because their mechanical properties can be easily tailored, they show minimal bioactivity due to the bio-inertness of PEG. Therefore, the bio-modification and copolymerization of PEG have become interesting strategies to modulate interactions with cells [8]. At the same time, the use of biopolymers (e.g., gelatin, chitosan, collagen, alginate, hyaluronic acid, and fibrinogen) as hydrogel precursors has gained an increased interest in the scientific community due to the superior biological functions of natural-based materials, such as cell adhesion and biodegradation [10,11]. Among them, gelatin has shown great potential [12,13,14]. Gelatin is a mixture of proteins derived from the acid or alkaline hydrolysis of collagen, and it contains specific peptide sequences for the recognition of integrin cell receptors, which are responsible for cell adhesion [15,16]. Nowadays, gelatin is widely used for TE purposes because of its excellent biocompatibility, biodegradability, low antigenicity, abundant availability in nature, and low cost [17]. Furthermore, a large variety of chemical modifications of the gelatin structure have been proposed for chemical hydrogels generation [18,19].

In this work, we investigated a different approach for the fabrication of photocurable poly(ethylene glycol)diacrylate (PEGDA) hydrogels, an approach that incorporates unmodified gelatin type A from porcine skin within the cross-linked gelatin network, using gelatin itself as the co-initiator in a Norrish type II radical photo-initiating system whereby camphorquinone (2,6-bornanedione, also known as CQ) is employed as photo-initiator.

**Figure 1:**
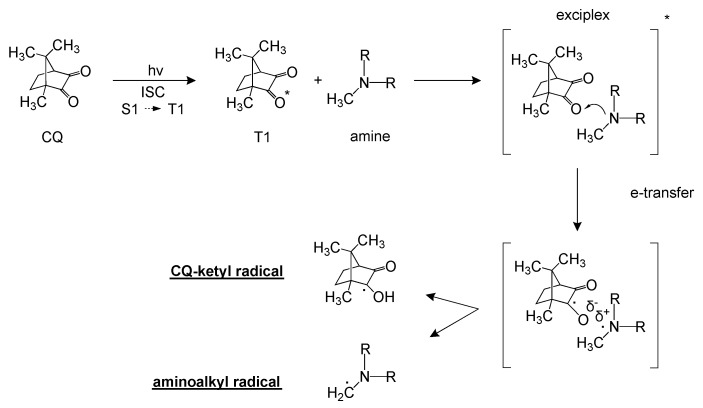
Key steps of free-radical photogeneration pathway of a camphorquinone (CQ)/amine system.

Camphorquinone is widely employed in visible-light initiating systems (absorption peak at 469 nm), especially for the preparation of photo-cross-linkable dental resins [20,21]. Several studies have demonstrated that the addition of a co-initiator, typically aliphatic or aromatic amines, leads to the generation of efficient photo-initiating systems [22,23,24]. The key steps of free-radical photogeneration pathway of a CQ/amine system are schematically reported in Figure 1.

According to the photochemistry of ketones, the absorption of a photon upon visible-light irradiation promotes the photoexcitation of camphorquinone into a singlet state (S1) due to the n,π* transition of the α-dicarbonyl chromophore [22,25,26]. This short-lived state (approximately 20 μs), may undergo non-radiative transitions, deactivate, decompose, or relax to a lower energetic triplet state (T1) by intersystem crossing (ISC). While in T1, CQ reacts with the amine to form an excited complex state known as “exciplex.” Within the exciplex, two radical ion species are generated by charge transfer from the nitrogen lone pair of the electron-donor amine to the activated carbonyl of CQ, which acts as the electron acceptor. Finally, if the amine species has any available carbon at the α-position of nitrogen, two free radicals are produced via intermolecular H-abstraction: a reactive aminoalkyl radical and a relatively unreactive camphorquinone ketyl radical. The aminoalkyl radicals thus generated are responsible for initiating the photopolymerization of a monomer, whereas the camphorquinone-ketyl radicals tend to deactivate or may also act as terminating agents [22,27,28,29,30,31].

In this study, we investigated the use of gelatin as the electron/proton donor species of the photo-initiating system instead of the traditional amines.

Accordingly, PEGDA-gelatin hydrogels were prepared by photopolymerization induced by visible light irradiation. The kinetics of the photopolymerization was studied by means of real-time photorheology, investigating the influence of gelatin on curing efficiency. The incorporation of gelatin within the cross-linked network was evaluated by using infrared spectroscopy. The thermo-mechanical performance, swelling behavior, and gel content of the resulting hydrogels were also studied.

## 2. Experimental Section

### 2.1. Materials

Gelatin (type A, Vetec reagent grade powder, gel strength = 300 g Bloom, *M_n_*= 50,000–100,000) from porcine skin, poly(ethylene glycol) diacrylate (PEGDA, *M_n_*= 700 g mol^−1^), camphorquinone (CQ, 97%) and Dulbecco′s phosphate buffered saline (DPBS, pH 7.0–7.3) were purchased from Sigma-Aldrich (Milano, Italy) and used as received without further purification. Deionized water (DIH_2_O) was obtained from a reverse osmosis (RO) purification system.

### 2.2. Precursor Formulations and Preparation of Hydrogels

PEGDA (35 wt%) and gelatin powder were dissolved in deionized H_2_O (DIH_2_O) at 60 °C under gentle stirring until the mixture became homogeneous. Different formulations were prepared while keeping the amount of PEGDA and CQ constant and while varying the gelatin ratio with respect to PEGDA (5, 10, and 15 wt%). The photo-initiator, previously dissolved in ethanol because of its poor water solubility, was then added to each precursor solution. The final compositions of the formulations are reported in Table 1. The formulation prepared without adding gelatin (PG0) was taken as reference.

The hydrogels were then prepared by casting the final formulations into homemade polydimethylsiloxane (PDMS) molds and irradiating for 8 min with visible light using a Hamamatsu LC8 lamp with a cutoff filter below 400 nm and that was equipped with an 8 mm light guide. The energy dose (8 mW/cm^2^) was periodically checked to guarantee the reproducibility of the photo-curing process by means of a C6080-04 Hamamatsu light power meter.

### 2.3. Photorheology

Real-time photorheological measurements were carried out to investigate the photopolymerization kinetics of the different formulations. The tests were performed using an Anton PAAR Modular Compact Rheometer (Physica MCR 302, Graz, Austria) in parallel-plate mode (25 mm diameter), and the visible-light source was provided by positioning the light guide of the Hamamatsu LC8 lamp under the bottom plate. During the measurements, the gap between the two glass plates was set to 0.3 mm, and the sample was kept under a constant shear frequency of 5 Hz. The irradiating light was switched on after 60 s to allow the system to stabilize before the onset of polymerization. According to preliminary amplitude sweep measurements, all of the tests were carried out in the linear viscoelastic region at a strain amplitude of 1%. The kinetics of photopolymerization was studied as a function of the changes in the shear modulus (G′) of the sample versus the exposure time. The measurements were repeated three times for each precursor formulation to verify reproducibility.

Additionally, amplitude sweep measurements (AS) were performed to determine the limit of the viscoelastic region of the different hydrogel samples. The AS tests were carried out at constant temperature (25 °C) from 0.1% to 100%, setting the shear frequency at 5 Hz.

### 2.4. Spectroscopic Characterization

Attenuated total reflectance-infrared spectroscopy (ATR-FTIR) was used to evaluate the chemical composition of the hydrogels. The experiments were conducted on dried samples by means of a Thermo Scientific Nicolet iS50 FTIR Spectrometer (Milano, Italy) equipped with a diamond crystal ATR accessory. Thirty two ATR spectra were collected with a resolution of 4 cm^−1^ in the range of 4000–600 cm^−1^ for each sample. The spectrum of physically cross-linked gelatin type A was taken as a reference. The results were acquired and evaluated using the Omnic software.

### 2.5. Dynamic Mechanical Thermal Analysis

The glass transition temperature of the cured hydrogel was investigated by means of dynamic mechanical thermal analyses (DMTAs). DMTA measurements were performed on dried thin and flat rectangular samples with a Tritec 2000 DMA (Triton Technology Ltd, London, UK). The tests were carried out between −80 and +100 °C, with the setting of a temperature ramp of 3 °C min^−1^ and the application of a force to the sample with a constant frequency of 1 HZ and 20 μm of displacement. The concomitant changes of G′ and tanδ were measured as functions of the temperature increase.

### 2.6. Swelling Degree and Gel Content

The swelling capability of the hydrogels in DPBS was measured gravimetrically at 37 °C. The samples, obtained following the procedure previously described, were first immersed in DIH_2_O at room temperature for 24 h to leach the unreacted-soluble fraction and/or the residues of CQ entrapped in the network, and they were then dried overnight in a vacuum oven (50 °C, 600 mbar). Then, the dried samples were immersed in DPBS at 37 °C. The swelling kinetics was determined after taking out the soaked samples from the DPBS solution at different time intervals and weighing them once the surface droplets were wiped off with wet paper. The samples were then placed back into DPBS, and the protocol was repeated until no further weight change was observed. The swelling degree (SD) was determined using the following equation:

$$\mathrm{SD}\left(\%\right)=100*\frac{W_{t}-W_{0}}{W_{0}}$$

where *W*_t_ is the weight of the hydrogel sample at a certain time and *W*_0_ is the weight of the dried sample recorded as the initial weight. 

The gel content was measured to evaluate the amount of the insoluble fraction of the cross-linked samples. The previously dried samples were held in a metal net, weighed, and then immersed in chloroform for 8 h at room temperature to dissolve the uncrosslinked polymer. The samples were then dried overnight in a vacuum oven (50 °C, 600 mbar) and weighed again. The gel content was determined as the weight difference before and after solvent extraction. The results of both the swelling degree and gel content were averaged over measurements carried out on three different samples for each hydrogel batch.

## 3. Results and Discussion

### 3.1. Photopolymerization Kinetics 

**Figure 2:**
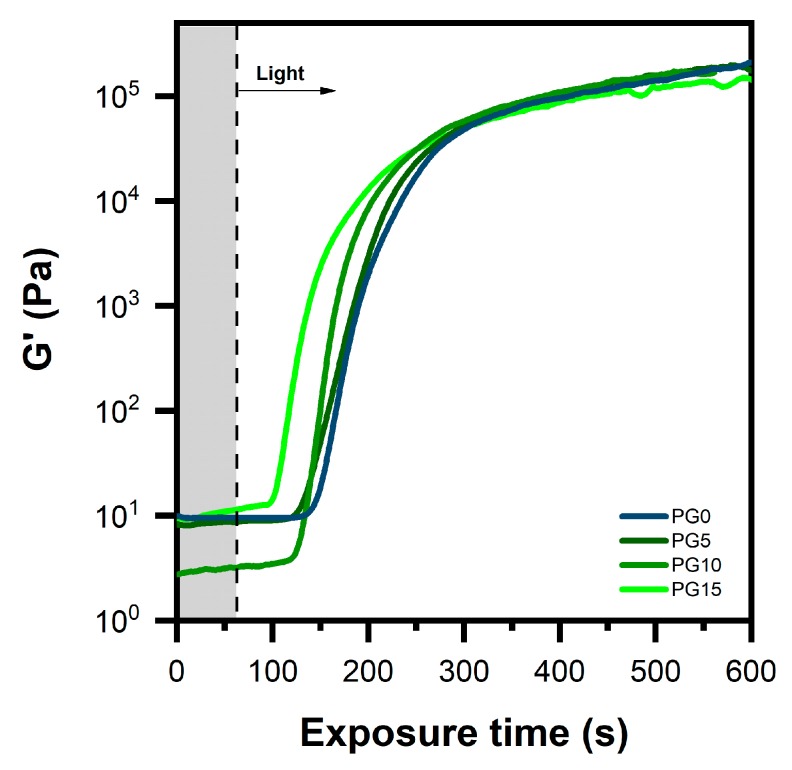
Photopolymerization kinetics of the different formulations (light switched on after 60″).

The influence of gelatin on the kinetics of PEGDA photopolymerization was evaluated by the means of photorheology. The formulation containing just camphorquinone as the initiating species (PG0) showed slow kinetics and a high exposure time (73 ± 3 s) to start the polymerization (Figure 2). This result can be attributed to the limited photo-initiating efficiency of CQ itself, which can initiate cross-linking without the addition of a co-initiator but only at a low rate [22]. Indeed, in the absence of an electron/hydrogen donor species, polymerization occurs by a chain reaction between the free radicals generated, via hydrogen abstraction, from the PEGDA monomers by the activated triplet state of CQ (T1). However, the low efficiency of this reaction was reflected by the slow kinetics of the polymerization reaction [27]. In contrast, all of the formulations containing gelatin (PG5, PG10 and PG15) showed decreased exposure times as the gelatin content increased (up to 34 ± 2 s for PG15), proving that gelatin can accelerate photopolymerization.

The reason for the faster photopolymerization of the PEGDA-gelatin formulations was the higher efficiency of the photo-initiating system. Differently from what was discussed before for PG0, primary radicals are produced via electron/proton transfer from the amine segments of gelatin by the activated triplet state of camphorquinone, which acts as electron/proton acceptor. These radicals rapidly approach the unsaturated bonds (C=C) of PEGDA monomers, generating new propagating radicals that initiate polymerization via a chain reaction. Furthermore, they remain covalently attached to the cross-linked network [32]. The intermolecular electron/proton transfer between CQ and its co-initiator is able to proceed faster in comparison to H-abstraction from PEGDA monomers [31]. As a consequence, the rate of photopolymerization was significantly increased.

### 3.2. Incorporation of Gelatin Within the Cross-Linked Network

The incorporation of gelatin segments within the cross-linked network of PEGDA was investigated by means of ATR-FTIR spectroscopy. The measurements were performed on dried samples, which had been previously washed for 6 hours with water to leach the unreacted-soluble fraction. Since gelatin molecules aggregate in water below 30–35 °C, thus generating a physical network that can be easily broken at higher temperature, the washing step was carried out at 60 °C to guarantee that the gelatin was chemically linked to the network and not just physically incorporated.

**Figure 3:**
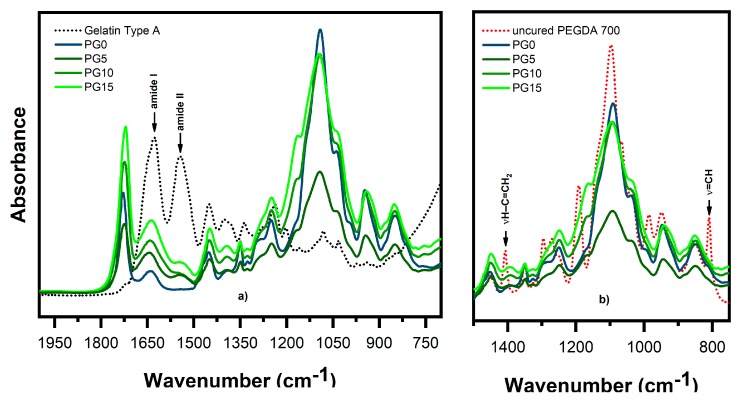
Attenuated total reflectance-infrared spectroscopy (ATR-FTIR) spectra of (**a**) the hydrogel samples and physically cross-linked gelatin reported in the range of 2000–700 cm^−1^; (**b**) a comparison between the spectra of the different cured samples and the spectrum of uncured poly(ethylene glycol) diacrylate (PEGDA) in the region 1950–1450 cm^−1^.

The ATR-FTIR spectra of the hydrogels, gelatin type A, and uncured PEGDA are reported in Figure 3.

The reference spectrum of physically cross-linked gelatin showed characteristic peaks at around 1630 cm^−1^ for the C=O stretching mode (amide I band) and 1530–1550 cm^−1^ for N–H deformation mode (amide II band) [15,17,32]. As illustrated in Figure 3a, the amide bands of gelatin could still be observed in all of the spectra of the hydrogels prepared by adding gelatin within the precursor formulation (PG5, PG10 and PG15). These findings confirm that the gelatin remained covalently attached to the cross-linked network of PEGDA after participating in the photogeneration of radicals upon reaction with CQ.

ATR-FTIR spectroscopy was also used to qualitatively confirm the curing process by evaluating the intensity of the decrease of the absorption bands corresponding to the C=C vibrations of acrylates as a result of monomer conversion. Figure 3b shows a comparison between the spectra of the different cured samples and the one of uncured PEGDA. Even if it was not possible to measure any difference in the intensity of the stretching vibration at 1630 cm^−1^ (vC=C) due to overlap with the gelatin amide I band, it could be seen that the C=C vibrations at 1412 cm^−1^ (vH–C=CH_2_) and 808 cm^−1^ (v=C–H) almost disappeared in all of the samples after irradiation. This indicates a high conversion of the double bonds during photocrosslinking. 

In order to confirm the crosslinking of the PEGDA-gelatin system, the weight percentage of the insoluble fraction of the polymers was evaluated after solvent extraction (Table 2). The PG0 sample presented a gel content of 90 wt%, confirming that, even in the absence of a co-initiator, a network can be formed; however, in this case, about 10 wt% of the monomers did not take part. All the PEGDA-gelatin hydrogels had a higher gel content (>95%), thus confirming an improved photo-crosslinking in the presence of gelatin.

### 3.3. Characterization of the Hydrogel

**Figure 4:**
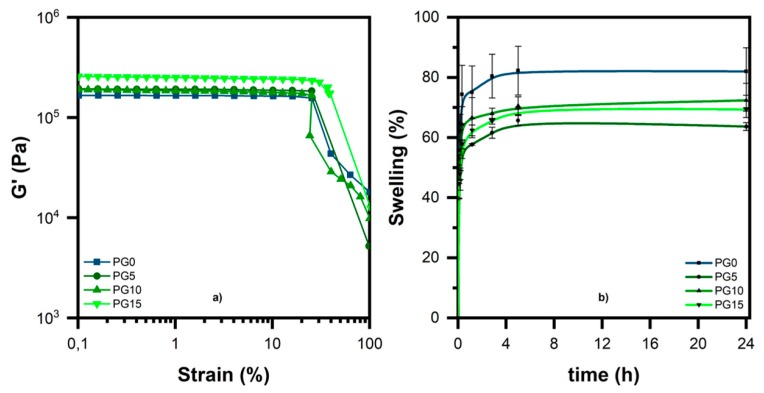
(**a**) Amplitude sweep curves and (**b**) swelling degree of the different hydrogel samples in Dulbecco′s phosphate buffered saline (DPBS) at 37 °C.

Amplitude sweep measurements were carried out on the freshly cross-linked hydrogels to evaluate their mechanical resistance over an increasing strain range (0.1%–100%). The results (Figure 4a) reveal that gelatin had no significant influence on the mechanical properties of the resulting hydrogels, since just the PG15 sample showed a slightly higher resistance under deformation, probably due to the generation of stronger cross-linked networks during photopolymerization. Essentially no variations were observed in terms of elastic modulus G′.

DMTA analyses were then performed on dried samples in order to evaluate the glass transition temperature of the cured networks (Table 2). The measurements showed a moderate increase in the glass transition temperatures (T*_g_*) for all of the PEGDA-gelatin samples (up to −35.6 °C in the case of PG15) compared to the one of PG0 (−42.2 °C). Once again, this suggests a slight strengthening of the cross-linked networks as a result of the more efficient polymerization in the presence of gelatin. 

The evaluation of the swelling ratio was used to characterize the absorption properties of the hydrogels. The tests were carried out in DPBS (pH 7.0–7.3) at 37 °C, in view of potential biomedical applications. The PEGDA-gelatin hydrogels showed moderate swelling ratios (Figure 4b and Table 2), varying in the range 60%–74% depending on the gelatin content. 

These values were slightly lower than the swelling degree reached by the sample prepared without gelatin (82%), indicating that the use of gelatin as a co-initiator led to the generation of higher cross-linked networks that were less prone to swelling. In addition, it could be observed that all of the samples showed fast swelling kinetics, because they absorbed the vast majority of water in the first 30 min, reaching swelling equilibrium after approximately 6 h (*t*_e_) when no further weight change was observed. There was no evidence of a specific trend in the absorption of DPBS with increasing gelatin content.

## 4. Conclusions

In this study, we developed a novel approach for the preparation of PEGDA-gelatin hydrogels using gelatin as the co-initiator of a radical photo-initiating system which employed camphorquinone as a Norrish type II photo-initiator. The real-time photorheological measurements confirmed that gelatin promoted the photopolymerization at any concentration, reducing the induction time of photo-crosslinking. ATR-FTIR spectroscopy proved that gelatin segments were incorporated within the network of PEGDA after taking part in the photogeneration of the radicals that reacted with CQ via intermolecular electron/proton transfer. The resulting PEGDA-gelatin hydrogels showed moderate swelling in DPBS (up to 74%) and a high gel content (>95%), suggesting that the use of gelatin led to the generation of stronger cross-linked networks. The limited influence of gelatin on the mechanical properties of the hydrogels could be justified by the fact that the 3D-network was essentially generated by PEGDA, whereas gelatin was just incorporated. Nevertheless, one might expect that gelatin should affect the mechanical behavior by acting as a plasticizer due to the length of its chain, but, simultaneously, it could offer more crosslinking points to generate a more packed network. These two effects could counter balance, thus giving a small variation of the final properties. 

## Figures and Tables

**Table 1:** Precursor formulations.

Formulation	PEGDA 700 (wt%)	Gelatin Type A ^a^ (wt%)	CQ^a^ (wt%)
PG0	35	0	2
PG5	35	5	2
PG10	35	10	2
PG15	35	15	2

^a^ The values refer to the amount of crosslinker (PEGDA 700).

**Table 2:** Hydrogel properties.

Formulation	T*_g_* (°C)	Swelling Ratio (%)	Gel Content [%]
**PG0**	−42.2	82 ± 9	90 ± 1.6
**PG5**	−37.5	60 ± 2	96 ± 0.6
**PG10**	−36.7	74 ± 8	95 ± 0.4
**PG15**	−35.6	69 ± 1	96 ± 0.3
